# Direct conversion of cellulose to l-lactic acid by a novel thermophilic *Caldicellulosiruptor* strain

**DOI:** 10.1186/s13068-022-02137-7

**Published:** 2022-05-02

**Authors:** Vitali A. Svetlitchnyi, Tatiana P. Svetlichnaya, Doris A. Falkenhan, Steve Swinnen, Daniela Knopp, Albrecht Läufer

**Affiliations:** BluCon Biotech GmbH, Nattermannallee 1, 50829 Cologne, Germany

**Keywords:** Anaerobic, *Caldicellulosiruptor*, Consolidated bioprocessing, l-Lactic acid, Thermophilic bacteria, High temperature, Lignocellulose

## Abstract

**Background:**

Consolidated bioprocessing (CBP) of lignocellulosic biomass to l-lactic acid using thermophilic cellulolytic/hemicellulolytic bacteria provides a promising solution for efficient lignocellulose conversion without the need for additional cellulolytic/hemicellulolytic enzymes. Most studies on the mesophilic and thermophilic CBP of lignocellulose to lactic acid concentrate on cultivation of non-cellulolytic mesophilic and thermophilic bacteria at temperatures of 30–55 °C with external addition of cellulases/hemicellulases for saccharification of substrates.

**Results:**

l-Lactic acid was generated by fermenting microcrystalline cellulose or lignocellulosic substrates with a novel thermophilic anaerobic bacterium *Caldicellulosiruptor* sp. DIB 104C without adding externally produced cellulolytic/hemicellulolytic enzymes. Selection of this novel bacterium strain for lactic acid production is described as well as the adaptive evolution towards increasing the l-lactic acid concentration from 6 to 70 g/l on microcrystalline cellulose. The evolved strains grown on microcrystalline cellulose show a maximum lactic acid production rate of 1.0 g/l*h and a lactic acid ratio in the total organic fermentation products of 96 wt%. The enantiomeric purity of the l-lactic acid generated is 99.4%. In addition, the lactic acid production by these strains on several other types of cellulose and lignocellulosic feedstocks is also reported.

**Conclusions:**

The evolved strains originating from *Caldicellulosiruptor* sp. DIB 104C were capable of producing unexpectedly large amounts of l-lactic acid from microcrystalline cellulose in fermenters. These strains produce l-lactic acid also from lignocellulosic feedstocks and thus represent an ideal starting point for development of a highly integrated commercial l-lactic acid production process from such feedstocks.

## Background

l-Lactic acid is a platform chemical widely used in bioplastic, food, feed, chemical, textile and pharmaceutical industries. The demand for l-lactic acid is growing steadily because significant part of lactic acid produced is used as a monomer for the synthesis of the biobased, biocompatible and biodegradable polymer polylactic acid (PLA) [1–3]. PLA is generally considered as one of the most promising bioplastics for the substitution of petrochemical-based plastics, mainly due to its easy processability and favorable mechanical properties [1, 4–6]. PLA applications range from medical (surgical sutures, tissue regeneration) and agricultural (mulch films, bags) products to packaging and single-use plastics (cups, cutlery). The application of PLA for the production of single-use plastics has gained particularly interest in the last years as these plastics are among the largest contributors to environmental pollution [7]. Facing this huge pollution problem along with the depletion of fossil fuels and the increasing emission of carbon dioxide, bioplastics such as PLA are a promising solution towards a sustainable society [1, 8]. The global lactic acid demand is expected to increase from a market value of $2.1 billion in 2016 to $9.8 billion by 2025 [9]. By 2025, utilization of lactic acid for PLA production is expected to reach 50% of total lactic acid available on the global market [10]. Thus, ways of production of the building block l-lactic acid have to be found which are cost effective and ethically acceptable.

Today about 90% of l-lactic acid is produced from sugar or starchy biomass, posing a competition for the raw materials with food industry [2, 3]. This first-generation lactic acid is obtained by fermentation using mesophilic lactic acid bacteria of the genera *Lactobacillus, Lactococcus* and *Streptococcus*, thermophilic bacteria of the genus *Bacillus,* and fungi of the genus *Rhizopus* [2, 10, 11]. The raw materials for first-generation lactic acid production constitute 40–70% of the total production cost [11].

In the last decade research efforts were made to replace this first-generation l-lactic acid by the second-generation l-lactic acid made from lignocellulosic feedstocks, including pretreatment, enzymatic hydrolysis, sugar fermentation and process design [2, 11]. Fermentative processes with lignocellulosic feedstocks originating from agriculture, forestry or industry residues are highly desirable for lactic acid production, since these feedstocks are abundant, economically attractive and cannot be used as food components. However, cellulose and hemicellulose in lignocellulosic substrates cannot be directly utilized by lactic acid bacteria [12], *Bacillus* [13] and *Rhizopus* [14], which are currently used for first-generation l-lactic acid production.

Most of the second-generation processes that are under development towards industrial scale, involve pretreatment and addition of enzymes for cellulose and hemicellulose hydrolysis, production of C5 and C6 sugars and fermentation with organisms applied in the first-generation lactic acid production. These organisms utilize C6 sugars, but only few of them can ferment C5 sugars [2, 11, 15]. Achieving both an effective biomass hydrolysis and a complete sugar conversion are essential for an economic process. However, the high costs of externally added enzymes make the second-generation l-lactic acid from lignocellulose economically not attractive, as enzyme costs contribute nearly a quarter to the minimum l-lactic acid selling price [3].

A process strategy that aims to circumvent this critical cost-increasing item is the consolidated bioprocessing approach (CBP) [16–18]. An efficient CBP, i.e., direct biological conversion of pretreated lignocellulose without added enzymes, to l-lactic acid with the use of single species of microorganisms (monoculture) or combinations of different strains (co-cultures) in a single unit operation would be highly desirable [18].

In CBP, an organism or a mixed culture of organisms produces enzymes for hydrolysis of cellulose and hemicellulose in lignocellulosic biomass and ferments the C5 and C6 sugars into ethanol, lactic acid or other valuable products without addition of cellulolytic or hemicellulolytic enzymes. Several mesophilic and thermophilic cellulolytic and non-cellulolytic microorganisms with engineered cellulase activity are under development for the application in CBP [16, 19–22]. Until now, the most well-developed cellulolytic candidates for thermophilic CBP are the anaerobic thermophilic bacterium *Clostridium thermocellum* and the anaerobic thermophilic bacteria of the genus *Caldicellulosiruptor* [23–25].

Realization of a CBP at high temperatures (≥ 70 °C) would offer several advantages over mesophilic (15–45 °C) and moderately thermophilic (45–60 °C) conditions: no requirement for cooling, reduced risk of contamination, decreased medium viscosity, and elimination of pathogenic bacteria [11, 26].

Thermophilic bacteria of the genus *Caldicellulosiruptor* (showing a temperature optimum for growth ≥ 70 °C) are regarded as very promising CBP organisms [16, 22, 27]. *Caldicellulosiruptor* species effectively hydrolyze both cellulose and hemicelluloses, metabolize C5 and C6 sugars, and can grow on pretreated as well as untreated lignocellulosic materials like switchgrass, miscanthus, wheat straw, corn stalks, birch, poplar, and waste paper [22, 27–33].

*Caldicellulosiruptor* species generally produce acetate, lactate, ethanol, H_2_ and CO_2_ as fermentation products [22, 28–32]. *Caldicellulosiruptor* species have so far been investigated primarily for conversion of lignocellulose to H_2_ [29, 34, 35] and ethanol [22, 49].

In our previous work [22] we have isolated and characterized seven novel strains of *Caldicellulosiruptor*, which are phylogenetically closely related to *Caldicellulosiruptor saccharolyticus* [36]. All of them grew at 72 °C on C5 and C6 sugars and effectively degraded cellulose, xylan as well as pretreated lignocellulosic substrates like poplar, spruce, miscanthus, wheat straw, corn cobs, corn stalks, and sugar cane bagasse. Six of the isolated strains produced up to 3 g/l lactic acid as the main fermentation product when cultivated in flasks with cellulose, xylan, cellobiose, glucose or xylose as substrates.

The high growth temperature and hydrolytic capabilities of the isolated strains, the generation of lactic acid as the main fermentation product and the substantial concentrations of lactic acid reached in flask with strains grown without pH control suggested a great potential of these strains for production of lactic acid from lignocellulose in a CBP process without addition of external enzymes [22]. However, a significant improvement of the isolated wild-type *Caldicellulosiruptor* strains regarding lactic acid concentration, lactic acid ratio in fermentation products and production rate is required to establish a commercially viable CBP of lignocellulose to lactic acid.

The required improvement can be reached by adaptive laboratory evolution methods, which is a powerful technology for classical strain improvement [37, 38]. Adaptive evolution is a well-established method that enables the development of phenotypes in microbial strains by the application of long-term selection under specific growth conditions. As the resulting phenotypes can be directly connected to the applied growth conditions, adaptive evolution may enable to acquire insights in the molecular mechanisms determining the phenotype [37]. Adaptive evolution was already successfully applied for improvement of L-lactic acid production from sugars by *Thermoanaerobacterium* sp. [39] and of d-lactic acid production from sugars by *Leuconostoc mesenteroides* [40].

Here we present the results of selection and improvement by adaptive evolution of the wild-type strain *Caldicellulosiruptor* sp. DIB 104C [22] and generation of *Caldicellulosiruptor* strains and cultures with exceptionally high l-lactic acid production. Two evolved strains produced 70 g/l of l-lactic acid on microcrystalline cellulose as substrate compared to 6 g/l formed by the parent wild-type strain. The lactic acid ratio in fermentation products was 96 wt% and the l-lactic acid ratio in d, l-lactic acid produced was 99.4%. We also show that the evolved strains produce l-lactic acid from lignocellulosic substrates with a lactic acid ratio in fermentation products of 75 wt%. The l-lactic acid from microcrystalline cellulose and lignocellulosic substrates was obtained in a consolidated bioprocessing, without the addition of external enzymes.

## Results

### Selection of *Caldicellulosiruptor* sp. strain DIB 104C for lactic acid production from microcrystalline cellulose and lignocellulose

**Fig. 1:**
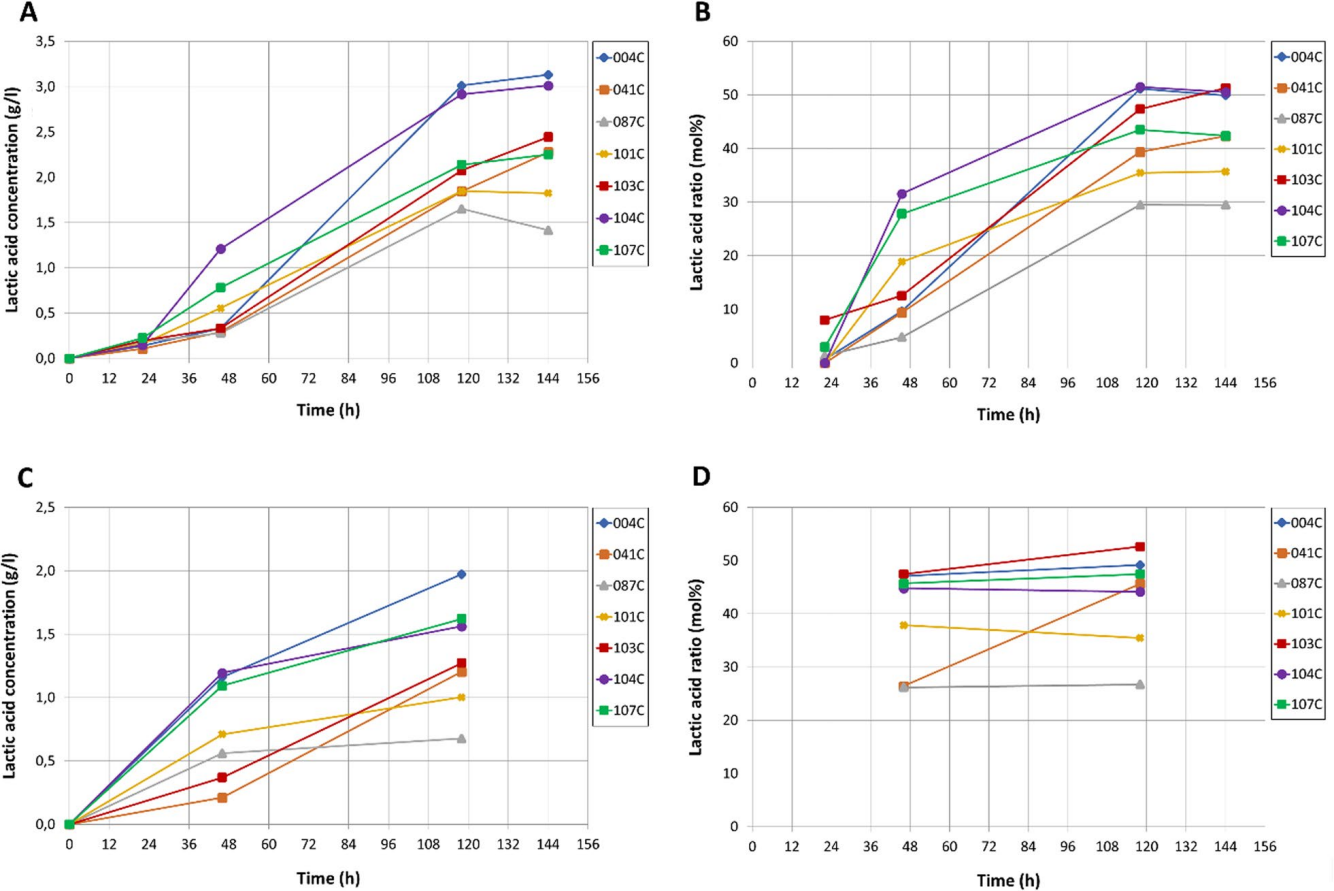
Lactic acid production by *Caldicellulosiruptor* strains DIB 004C, DIB 041C, DIB 087C, DIB 101C, DIB 103C, DIB 104C and DIB 107C. Bacteria were grown in flasks at 72 °C and 100 rpm on 10 g/l microcrystalline cellulose (**A**, **B**) or 5,9 g/l pretreated miscanthus (**C**, **D**). The media contained 10 g/l MOPS as buffer. Lactic acid concentration (g/l) (**A**, **C**) and lactic acid ratio (mol%) (**B**, **D**) in formed fermentation products (lactate + acetate + ethanol) are presented

The thermophilic cellulolytic and hemicellulolytic bacterium *Caldicellulosiruptor* sp. strain DIB 104C and 6 other *Caldicellulosiruptor* strains were previously isolated from environmental samples during a search for ethanol producers from lignocellulose [22]. The strain DIB 104C produced lactic acid as the main fermentation product as well as acetic acid and ethanol as minor fermentation products with cellulose, cellobiose, glucose, xylan and xylose as substrates [22]. Compared to the other 6 isolated *Caldicellulosiruptor* strains, DIB 104C displayed the highest lactic acid production rate (Fig. 1A, C) and a high lactic acid ratio in total organic fermentation products (lactate + acetate + ethanol) (Fig. 1B, D) when grown in flasks on microcrystalline cellulose (Avicel PH-101) or steam explosion pretreated miscanthus as substrates (Fig. 1). Therefore, the strain DIB 104C was selected for the development of a single-step lactic acid production technology from microcrystalline cellulose and lignocellulose.

### Improvement of lactic acid production of strain DIB 104C by adaptive laboratory evolution

**Fig. 2:**
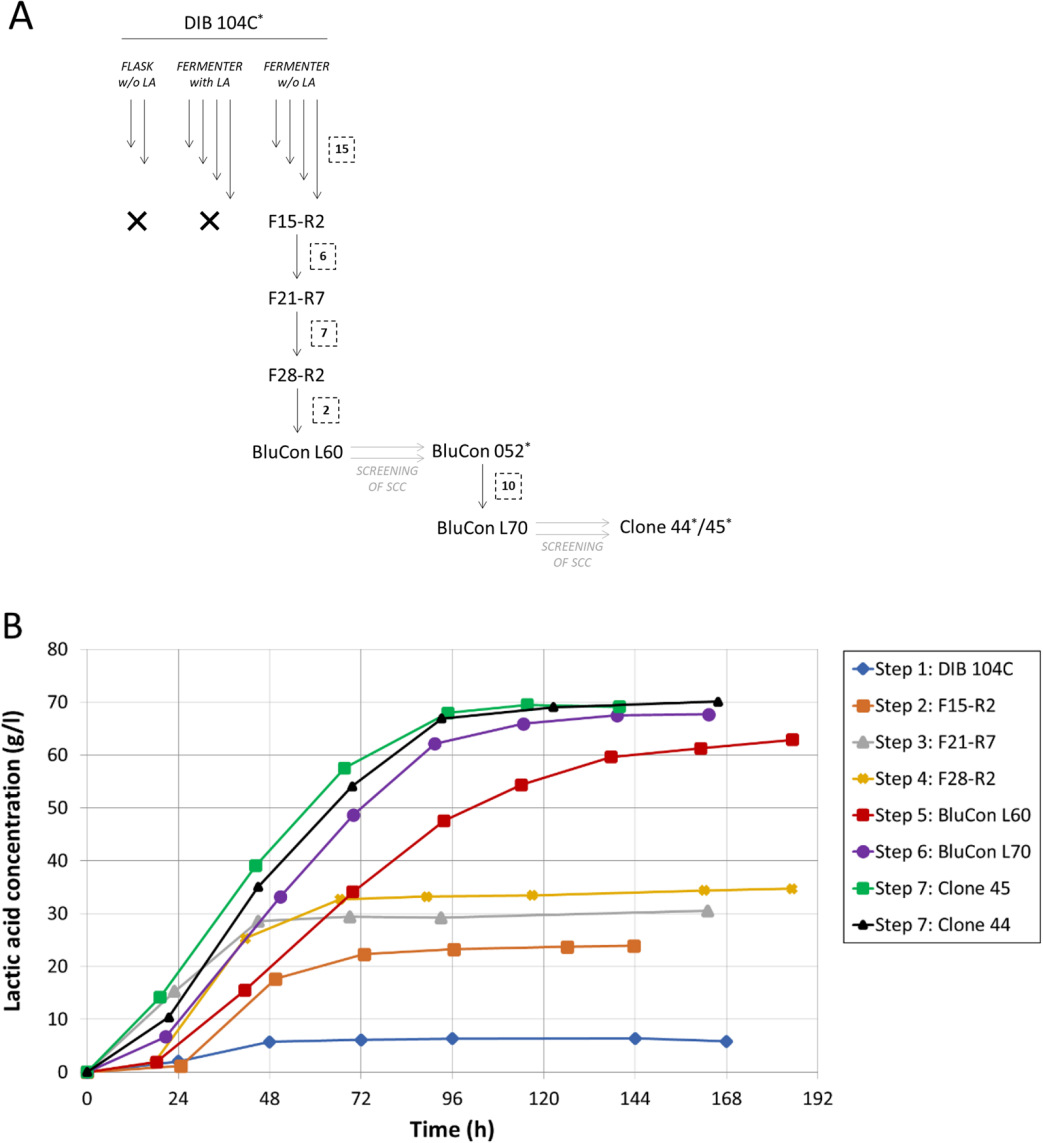
Lactic acid production by *Caldicellulosiruptor* DIB 104C cultures generated during adaptive evolution. **A** Strain DIB 104C was applied in sequential repeated batch transfers using media containing microcrystalline cellulose as substrate and with or without externally added lactic acid as stress factor. The transfers in flasks without externally added lactic acid (referred to as “w/o LA”) and in fermenters with externally added lactic acid (referred to as “with LA”) were not continued (indicated by the symbol “x”) because the improvement in lactic acid production was lower as compared to the transfers in fermenters without externally added lactic acid. The numbers of sequential transfers in fermenters are shown in dotted line boxes. Occasionally during the repeated batch transfers, a significant increase in lactic acid production was observed (referred to as “steps”) and an aliquot of the respective cultures was stored for preservation and isolation of single-cell colonies (referred to as “SCC”). Culture names with an asterisk represent pure, isogenic strains; culture names without an asterisk represent mixed populations. **B** The mixed populations and isogenic strains with significantly improved lactic acid production (referred to as steps 1 to 7) were grown in fermenters on up to 200 g/l microcrystalline cellulose at 70 °C with pH stabilized at 6.4. Lactic acid production with cultures from evolution steps 1 to 7 are presented

During growth in fermenters on microcrystalline cellulose, up to 6 g/l lactic acid was formed by the wild-type strain DIB 104C (Fig. 2B, step 1). The maximum lactic acid production rate was 0.15 g/l*h, the lactic acid ratio in the total fermentation products (lactate + acetate + ethanol) was 53 wt%, and the l-lactic content in total d, l-lactic acid formed (D + L enantiomer) was 97.4%. The wild-type strain DIB 104C was subjected to adaptive evolution with the aim to increase the resistance of bacteria to elevated lactic acid concentrations. Ten independent lines of evolution were applied: two lines of sequential transfers (repeated batch) in flasks and eight lines of sequential transfers in fermenters, either in the absence or in the presence of steadily increasing concentrations of externally added lactic acid (up to 30 g/l) (Fig. 2A). After the transfers, cultures were tested for lactic acid production by using flask assays with microcrystalline cellulose as substrate and CaCO_3_ as buffering component. Cultures obtained from flasks and from fermenters with externally added lactic acid did not show any improvement in lactic acid production, while cultures obtained from fermenters without externally added lactic acid did show improvement (data not shown). One selected culture showing the highest lactic acid production in terms of concentration (referred to as F15-R2, Fig. 2A, B) was used for further adaptive evolution in fermenters by repeated batch. The stepwise improvement of the selected culture is exemplified in the steady increase of the lactic acid production of cultures F21-R7 and F28-R2 (Fig. 2A, B, steps 2–4).

An improved culture designated BluCon L60 was obtained in one line of evolution after 30 sequential transfers in fermenters. The culture produced in fermenters up to 63 g/l lactic acid in 96 h when grown on 100 g/l microcrystalline cellulose (Fig. 2B, step 5). BluCon L60 displayed a maximum lactic acid production rate of 0.66 g/l*h, a lactic acid ratio in the total fermentation products of 98 wt% and a l-lactic acid content in total d, l-lactic acid formed of 99.4%. More than 150 single-cell colonies were isolated from BluCon L60 on solid agar medium in roll tubes containing amorphous cellulose as substrate. The isolated clones were selected on the formation of large clearing zones in cellulose suggesting high cellulase activity. All clones were tested for lactic acid production in flask assays with microcrystalline cellulose and CaCO_3_ (data not shown). Out of the 150 clones that were tested, the best ten reaching the highest lactic acid concentrations in flasks were selected (one of which is strain BluCon 052) and subjected to further independent evolution by repeated batch in fermenters (Fig. 2A).

After ten sequential repeated batch transfers in fermenters, a culture evolved from the clone BluCon 052 produced 70 g/l lactic acid from microcrystalline cellulose (Fig. 2B, step 6). The culture, designated BluCon L70, was streaked for single-cell colonies on plates in the presence of externally added lactic acid (25 g/l). Fifty clones showing growth were tested for lactic acid production in flasks on microcrystalline cellulose (data not shown). The best five reaching the highest lactic acid concentrations in flasks were selected and evaluated in fermenters. Two promising clones were identified (designated clone 44 and 45) that displayed faster growth as compared to the initial culture BluCon L70 and produced in fermenters up to 70 g/l lactic acid (68 g/l after 95 h cultivation), with microcrystalline cellulose as substrate (Fig. 2A, B, step 7). Both clones showed a maximum lactic acid production rate of 1.0 g/l*h, a lactic acid ratio in the total fermentation products of 96 wt%, and a l-lactic acid content in total d, l-lactic acid formed of 99.4%.

**Fig. 3:**
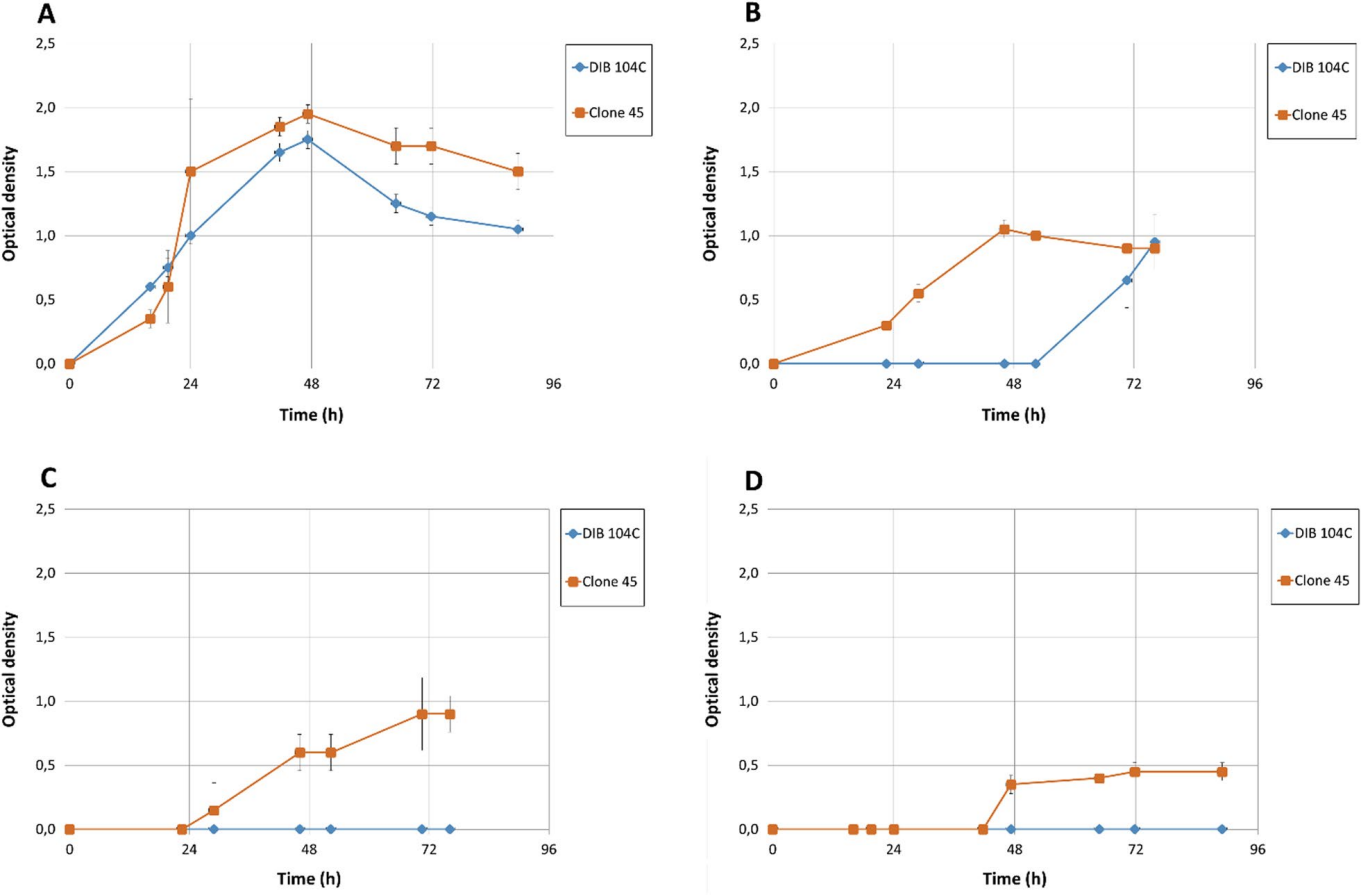
Effect of externally added lactic acid on growth of *Caldicellulosiruptor* DIB 104C wild-type strain and evolved strain clone 45. Bacteria were grown at 70 °C in 16 ml Hungate tubes with 9 ml medium containing 5 g/l glucose and 10 g/l MOPS. Lactic acid was added as sodium lactate to initial concentrations of 4.0 g/l (**A**), 15.5 g/l (**B**), 19.2 g/l (**C**) or 22.8 g/l (**D**). Growth was monitored by measurement of the optical density (McFarland units) of cultures directly in tubes using a densitometer DEN-1. One McFarland unit corresponds to an optical density OD_550_ of 1 when measured in 1-cm cuvettes

The adapted clone 45 showed higher tolerance to lactic acid compared to the initial wild-type strain DIB 104C when lactic acid was added to the medium before inoculation (Fig. 3). There were no differences in growth when the initial lactic acid concentration was 4 g/l (Fig. 3A). At 15.5 g/l lactic acid, clone 45 started to grow without a lag-phase, whereas the wild-type strain started to grow only after a lag-phase of 71 h (Fig. 3B). The adapted strain clone 45 grew at 19.2 g/l lactic acid (Fig. 3C) and at 22.8 g/l lactic acid (Fig. 3D) after a lag-phase of 24 h and 42 h, respectively. Wild-type strain DIB 104C did not show growth at these lactic acid concentrations (Fig. 3C, D).

Analysis of the fermentation broth showed that the microcrystalline cellulose provided in limiting concentrations was completely utilized by the evolved cultures. For example, during cultivation of BluCon L70 on 50 g/l microcrystalline cellulose in fermenters, the yield of lactic acid from the provided cellulose was 84.6% (from the maximum expected concentration of fermentation products at 100% utilization of the substrate) and the yield of total fermentation products (lactate + acetate + ethanol) was 93.5%.

### Fermentation products of wild-type strain DIB 104C and improved cultures thereof

**Fig. 4:**
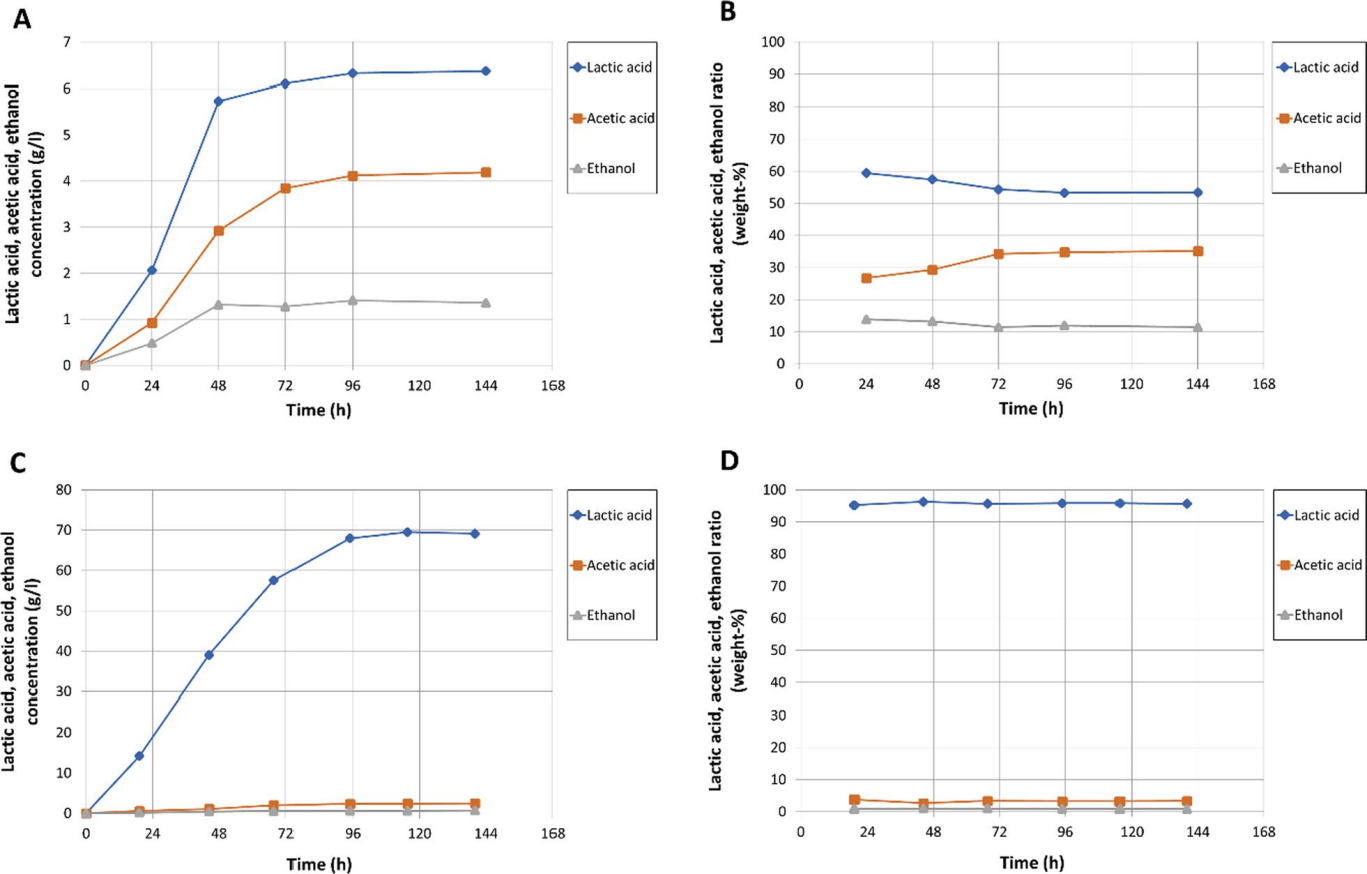
Lactic acid, acetic acid and ethanol production by *Caldicellulosiruptor* DIB 104C wild-type strain and evolved strain clone 45. Wild-type strain (**A**, **B**) and evolved clone 45 (**C**, **D**) were grown in fermenters on microcrystalline cellulose at 70 °C with pH stabilized at 6.4. Lactic acid, acetic acid and ethanol concentrations (g/l) (**A**, **C**) and products ratio (wt%) (**B**, **D**) in formed fermentation products (lactate + acetate + ethanol) are presented

Lactic acid, acetic acid and ethanol were the organic fermentation products, and lactic acid was the main fermentation product of the wild-type strain DIB 104C and all evolved cultures and clones during growth on microcrystalline cellulose as substrate. After adaptive evolution of the wild-type strain DIB 104C, the final lactic acid concentration increased 11 times from maximum 6.4 g/l for wild-type DIB 104C to 70 g/l for the evolved clone 45 (Fig. 4A, C), while the maximum rate of lactic acid production increased 6.7 times from 0.15 to 1.0 g/l*h. The ratio of lactic acid in the total fermentation products (lactate + acetate + ethanol) increased from 53 wt% for DIB 104C to 96 wt% for the evolved clone 45 (Fig. 4B, D). The ratio of acetate in the total fermentation products decreased from 35 wt% for DIB 104C to 3 wt% for clone 45. The ratio of ethanol in the total fermentation products decreased from 12 wt% for DIB 104C to 1 wt% for clone 45 (Fig. 4B, D).

### Lactic acid production from different lignocellulosic substrates by improved cultures

In our previous work, the growth of the wild-type strain DIB 104C on cellulose, cellobiose, glucose, xylan and xylose was documented [22]. In the current study, different types of complex substrates (including lignocellulose) were converted to lactic acid by the improved culture BluCon L60 (Table 1). Growth was observed at all substrate concentrations tested, and all substrates were converted to lactic acid as the main fermentation product. Lactic acid yields (conversion of C5 and C6 sugar polymers present in these substrates to lactic acid) upon growth in flasks at limiting substrate concentrations of 18–27 g/l were dependent on the substrate, with the highest values for microcrystalline cellulose (86%) and the lowest values for untreated wheat straw (26%), which was the most complex substrate tested (Table 1).

**Table 1:** Conversion of substrates to lactic acid by *Caldicellulosiruptor* BluCon L60

Substrate	Substrate concentration in the culture	Lactic acid produced	Lactic acid ratio in fermentation products	Lactic acid yield. C5/C6-sugars conversion to lactic acid	LAE yield. C5/C6-sugars conversion to lactate + acetate + ethanol
	g (dry) / liter culture	g/l	wt%	% of maximum	% of maximum
Microcrystalline cellulose Avicel	18.3	16.8 ± 0.5	92.4 ± 0.4	85.7 ± 2.7	96.9 ± 2.9
	27.4	24.6 ± 0,2	93.0 ± 0.2	83.7 ± 0.6	93.6 ± 0.5
Microcrystalline cellulose Jelucel OF30	18.0	16.6 ± 0.2	92.6 ± 0.2	85.1 ± 0.9	95.6 ± 1.2
	27.0	23.3 ± 0.3	93.5 ± 0.0	79.9 ± 1.1	88.5 ± 1.3
Filter paper (Whatman #1)	18.5	17.1 ± 0.2	92.9 ± 0.1	87.1 ± 1.0	97.6 ± 1.0
	27.8	24.5 ± 0.2	92.9 ± 0.2	83.2 ± 0.8	93.1 ± 0.8
Newspaper	18.4	8.1 ± 0,1	89.9 ± 0.2	58.9 ± 0.8	69.2 ± 1.1
	27.6	10.9 ± 1.0	87.8 ± 4.5	52.6 ± 5.1	64.4 ± 1.5
Cardboard (brown)	18.3	9.3 ± 0.4	88.4 ± 0.1	62.3 ± 3.0	75.1 ± 3.7
	27.4	10.4 ± 0.7	87.6 ± 0.5	46.5 ± 3.0	56.7 ± 3.4
Recycled paper	18.3	8.3 ± 0.2	89.0 ± 0.3	63.2 ± 1.1	75.4 ± 1.2
	27.4	11.9 ± 0.3	90.1 ± 0.2	60.3 ± 1.4	70.7 ± 1.6
Xylan from beechwood	18.1	13.6 ± 0.9	88.5 ± 0.2	73.1 ± 4.6	88.7 ± 5.6
	27.2	18.7 ± 0.6	89.1 ± 0.6	67.2 ± 2.0	80.6 ± 3.2
Starch (soluble)	17.2	14.4 ± 0.9	91.2 ± 0.6	75.1 ± 4.7	86.6 ± 5.7
	25.9	23.2 ± 0.3	93.0 ± 0.3	80.9 ± 0.9	90.4 ± 0.8
Wheat straw (not pretreated)	18.1	3.1 ± 0.2	75.2 ± 1.0	26.3 ± 1.8	39.6 ± 2.0
	27.1	4.4 ± 0.1	77.7 ± 0.1	24.3 ± 0.6	35.0 ± 0.8
Wheat straw (6% NaOH-refiner)	21.4	8.8 ± 0.6	79.4 ± 4.7	49.3 ± 3.5	71.0 ± 1.3

Substrate concentrations between 18 and 80 g/l were applied and a CaCO_3_ concentration of 50 g/l was added as buffer. Cultures were grown in flasks at 70 °C without shaking for a total time of 14 days to make sure that fermentation was performed until completion

## Discussion

The harmful effect of the fossil-based chemical industry on the environment has provoked a shift towards a bio-based industry that relies on microorganisms as catalysts. Some of the bio-based products are already well-established and even dominate global production, with bio-ethanol production by the yeast *Saccharomyces cerevisiae* as the flagship example [42]. Industrial microorganisms for the production of bio-based products have been selected for best performance, nevertheless, there is always room for further improvement of the product concentration, production rate and yield. In this regard, adaptive laboratory evolution has proven to be a successful method for the improvement of industrially relevant phenotypes [37, 38].

In this study, the thermophilic and cellulolytic bacterium *Caldicellulosiruptor* sp. strain DIB 104C [22] was selected as potential producer of lactic acid from lignocellulosic substrates (Fig. 1) and subjected to adaptive evolution to improve the lactic acid production (Fig. 2). In the course of the adaptive evolution process, the lactic acid concentration reached by the strain DIB 104C steadily increased 11-fold from 6 to 70 g/l on microcrystalline cellulose (Avicel PH-101) as substrate (Fig. 2). We emphasize that the concentration of 70 g/l was achieved without the addition of cellulases to the fermentation broth, which are required to hydrolyze the available cellulose into sugars that can be taken up and metabolized by DIB 104C. Besides improvement of lactic acid concentration, the evolved clone 45 showed an almost sevenfold improvement of the maximum lactic acid production rate, which increased from 0.15 to 1.0 g/l*h (Figs. 2, 4). Lactic acid ratio in the fermentation products rose significantly from 53 wt% in the wild-type strain DIB 104C to 96 wt% in clone 45 (Fig. 4). Lactic acid was the main fermentation product on microcrystalline cellulose with up to 99.8% of l-lactic acid in total d, l-lactic acid formed.

Evolved cultures fermented the sugars glucose, cellobiose, sucrose, xylose, arabinose and lactose to lactic acid as the main fermentation product. All lignocellulosic substrates tested, including wheat straw, were also fermented to lactic acid as the main fermentation product without the addition of external cellulases and xylanases (Table 1). Starch was also easily fermented to lactic acid (Table 1).

The evolved cultures and clones derived from *Caldicellulosiruptor* sp. DIB 104C are excellent candidates for the use in single-step l-lactic acid production from lignocellulosic substrates. They grow on C6-polymer cellulose, C5-polymer hemicellulose (xylan), various lignocellulosic substrates and convert the liberated C5 and C6 sugars to lactic acid. The bacteria display high lactic acid tolerance (Fig. 3) and at the moment can produce up to 70 g/l lactic acid on microcrystalline cellulose. No additions of external cellulases and hemicellulases are required to ferment lignocellulose to lactic acid.

Although several genera and species of lactic acid bacteria, *Bacillus* and fungi have been shown to produce generally between 30 and 100 g/l lactic acid on hydrolysates of lignocellulosic substrates [2, 12, 15], they require the addition of expensive cellulolytic and hemicellulolytic enzymes for substrate hydrolysis, which is still not an economically viable option for industrial applications.

The high lactic acid concentrations and maximum production rates reported here (70 g/l lactic acid and 1.0 g/l*h, respectively) were obtained with evolved strains originating from *Caldicellulosiruptor* sp. strain DIB 104C grown on microcrystalline cellulose. These values are similar to lactic acid levels and production rates reported for most of the non-cellulolytic lactic acid producing microorganisms during growth on lignocellulosic substrates pretreated and hydrolyzed by externally added enzymes: various lactic acid bacteria, 7–93 g/l and 0.2–3.0 g/l*h [12, 15]; *Bacillus coagulans*, 40–134 g/l and 0.7–2.5 g/l*h [13, 21]; and *Rhizopus oryzae*, 34–60 g/l and 0.7–1.0 g/l*h [2]. Consolidated bioprocessing of microcrystalline cellulose and pretreated beech wood with co-cultures of cellulase producing *Trichoderma reesei* and lactic acid producing *Lactobacillus pentosus* yielded 19.8–34.7 g/l lactic acid with a rate of 0.1–0.16 g/l*h [43]. Higher lactic acid concentrations and production rates were reached during growth on sugars or starch hydrolysates, e.g., *Enterococcus faecium* S156 on glucose (126 g/l and 5.25 g/l*h), *Lactobacillus paracasei* LA104 on fresh sweet potato (198 g/l and 3.83 g/l*h) [2], *B. coagulans* WCP10-4 on glucose (210 g/l and 3.5 g/l*h), and *B. coagulans* C106 on xylose (141 g/l and 4.8 g/l*h) [13].

Further work is needed to understand the mechanisms of the higher lactic acid production in the evolved strains. A first clue was obtained by performing a test for growth in the presence of externally added lactic acid (Fig. 3), which showed that the evolved clone 45 has a higher tolerance to lactic acid as compared to the wild-type strain DIB 104C (Fig. 3). Further clues could be obtained from methods such as whole-genome resequencing and genetic mapping to identify the mutations determining the higher lactic acid production and tolerance [39, 40]. Knowledge about the underlying mechanisms and genetic determinants are a powerful base for further process intensification by rational genetic engineering.

Further steps of adaptive evolution of *Caldicellulosiruptor* clone 45 are ongoing to further increase the lactic acid concentration, tolerance, and production rate. In addition, optimization of lignocellulose pretreatment to make sugar polymers more accessible for secreted enzymes, is an important part in the development of single-step lignocellulosic lactic acid production [44].

The results of this study show that the inherent physiological properties of *Caldicellulosiruptor* make it a promising candidate for industrial lactic acid production and may advance the transition from a first-generation to an economically viable second-generation lactic acid production [45–47]. The *Caldicellulosiruptor* strain evolved in this study is able to use a diverse range of lignocellulosic feedstocks, such as paper sludge, wheat straw and others [22], which are relevant substrates of low value and high availability for the production of second-generation bio-products. The fact that *Caldicellulosiruptor* produces a vast range of cellulases and hemicellulases [27] is a considerable advantage as compared to other non-cellulolytic and non-xylanolytic lactic acid producing microorganisms, which require the addition of costly external enzymes for the hydrolysis of lignocellulosic substrates into fermentable sugars [2, 3, 12, 15].

## Conclusions

Here, we show for the first time that the adaptively evolved thermophilic strains of *Caldicellulosiruptor* are capable of producing up to 70 g/l l-lactic acid from microcrystalline cellulose as substrate. In addition, we show that l-lactic acid is also the major fermentation product when lignocellulosic substrates are used. No external enzyme additions were required since the appropriate cellulolytic and hemicellulolytic enzymes were provided by the bacteria. Therefore, these bacteria are promising starting points for the development of CBP processes aiming at the direct fermentation of lignocellulosic biomass to l-lactic acid.

## Methods

### Bacterial strains

The previously isolated strains of thermophilic anaerobic cellulolytic bacteria *Caldicellulosiruptor* sp. DIB 004C, DIB 041C, DIB 087C, DIB 101C, DIB 103C, DIB 104C and DIB 107C were used in this study [22].

### Cultivation

A prereduced modified medium described before [22] was used for cultivation of strains, evolved cultures and clones. Modifications included an increase of the NH_4_Cl concentration from 1.0 to 2.0 g/l and a substitution of Na_2_S by cysteine-hydrochloride. The medium contained (per liter of deionized water): K_2_HPO_4_, 1.5 g; KH_2_PO_4_, 3 g; MgSO_4_ × 7 H_2_O, 0.3 g; CaCO_3_ × 2 H_2_O, 0.05 g; NH_4_Cl, 2.0 g; NaCl, 0.5 g; NaHCO_3_, 0.5 g; NiCl_2_ × 6 H_2_O, 2 mg; FeSO_4_ × 7 H_2_O, 1 mg; NH_4_Fe(III) citrate, 10 mg; MnSO_4_ x H_2_O, 5 mg; CoCl_2_ × 6 H_2_O, 1 mg; ZnSO_4_ × 7 H_2_O, 1 mg; CuSO_4_ × 5 H_2_O, 0.1 mg; H_3_BO_4_, 0.1 mg; Na_2_MoO_4_ × 2 H_2_O, 0.1 mg; Na_2_SeO_3_ × 5 H_2_O, 0.2 mg; Na_2_WoO_4_ × 2 H_2_O, 0.1 mg; nicotinic acid, 2 mg; cyanocobalamin, 0.25 mg; p-aminobenzoic acid, 0.25 mg; calcium pantothenate, 0.25 mg; thiamine-hydrochloride, 0.25 mg; riboflavin, 0.25 mg; lipoic acid, 0.25 mg; folic acid, 0.1 mg; biotin, 0.1 mg; pyridoxine-hydrochloride, 0.1 mg; yeast extract (Difco), 0.5 g; resazurin, 0.5 mg; cysteine-hydrochloride, 1.0 g.

The medium was prepared under anaerobic conditions under O_2_-free N_2_ and pH was adjusted to 7.2. The medium was dispensed into flasks and Hungate tubes under N_2_ gas flow and afterwards was sterilized by autoclaving.

For precultures in Hungate tubes, 4.3 g/l of cellulose (strips of filter paper Whatman No. 1) was used as substrate. Tubes with 9 ml medium were incubated at 70 °C without shaking.

For precultures in 250 ml flasks, 10 g/l microcrystalline cellulose (Avicel PH-101, Sigma-Aldrich) was used as substrate and 10 g/l MOPS was added to increase buffering capacity. Flasks with 100 ml medium were incubated at 70 °C with shaking at 130 rpm.

For adaptive evolution and analysis of lactic acid production by evolved cultures in 110 ml flasks, 120 g/l microcrystalline cellulose (Avicel PH-101) was used as substrate and 50 g/l CaCO_3_ was applied as buffer. Flasks with 10 ml medium were incubated at 70 °C without shaking.

For analysis of substrate conversion in 110 ml flasks, up to 80 g/l (dry matter) of different lignocellulosic materials (microcrystalline cellulose, filter paper Whatman No. 1, newspaper “Kölner Stadtanzeiger”, cardboard from a packaging box, recycled paper from a paper plant, beechwood xylan from Carl Roth GmbH, soluble starch from Carl Roth GmbH, wheat straw from a local supplier) were used as substrate and 50 g/l CaCO_3_ was applied as buffer. Flasks with 10 ml medium were incubated at 70 °C without shaking.

Improved evolved strains were isolated as single-cell colonies from serial dilutions in Hungate roll tubes [48] with 30 g/l agar and 5 g/l acid-swollen amorphous cellulose or from serial dilutions on plates with 30 g/l agar, 20 g/l cellobiose, 20 g/l CaCO_3_ and 25 g/l lactic acid.

Growth of bacteria was monitored by analysis of fermentation products and determination of optical density (OD_600_) of the cultures. For the separation of cells from insoluble substrates, samples were centrifuged in 2 ml tubes for 20 s at 4000*g*.

Effect of externally added lactic acid on the growth of the wild-type strain DIB 104C and of the adapted strain clone 45 was investigated by cultivation of bacteria in 16 ml Hungate tubes at 70 °C without shaking. The tubes contained 9 ml medium with 5 g/l glucose as substrate and 10 g/l MOPS. Lactic acid was added to the medium from a sterile-filtered anoxic concentrated l( +)-sodium lactate solution (AppliChem). Growth of bacteria was monitored by measurement of the optical density (McFarland units) of cultures directly in tubes using a densitometer DEN-1 (Grant Instruments Ltd).

Miscanthus was pretreated by first-stage hydrolysis (2% (w/w) sulfurous acid, 150 °C, 45 min) followed by washing and second-stage steam explosion (190 °C, 5 min). Wheat straw was pretreated by hydrolysis (6% (w/w) NaOH, 85 °C, 120 min) followed by washing and refining. Substrates were dried at 45 °C for 72 h and used for growth experiments.

Composition of lignocellulosic substrates was determined according to the laboratory analytical procedure "Determination of structural carbohydrates and lignin in biomass" from National Renewable Energy Laboratory (NREL) [41]. Dry weight of substrates was determined after drying at 105 °C for 24 h.

Fermentations were carried out in 3-L stirred vessel fermenters (bbi-biotech GmbH, Berlin, Germany) with a working volume of 2 L. All vessels were equipped with double jackets for temperature control, two Rushton-type stirrer blades and pH-control loops. In order to maintain a constant pressure throughout the cultivation, vessels were additionally equipped with high-precision blow-off valves, controlling the pressure in the range of 1.3–1.5 bar. The medium as described above, except vitamins, was supplemented with up to 200 g/l microcrystalline cellulose. The medium was set to pH 6.4 by automatic addition of NaOH or Ca(OH)_2_ solution and this value was maintained throughout the fermentation run. In order to remove oxygen from the medium, the fermenter vessel was flushed with nitrogen for 1 h at a rate of 1 l/min; then cysteine was added as described above while gas flushing was stopped. Each fermenter was inoculated with 100 ml of preculture prepared as described above. A temperature of 70 °C was maintained during the entire fermentation run.

### Adaptive evolution

Adaptive evolution of wild-type strain DIB 104C was performed by sequential transfers (repeated batch) in flasks (two independent lines of evolution) and fermenters (eight independent lines of evolution). Flasks (110 ml) containing 10 ml medium with 120 g/l microcrystalline cellulose and 50 g/l CaCO_3_ were reinoculated every 7 days with 1 ml culture from previous transfer. Fermenters containing 2-L culture at the end of fermentation were subjected to repeated batch after 7 days of fermentation: 1.5 L of grown culture was removed and 1 L of fresh medium was added to the remaining 0.5-L culture. In some lines of evolution in flasks and fermenters up to 30 g/l lactic acid was added at the beginning of cultivation.

Cultures after transfers in flasks and fermenters were analyzed for lactic acid production in flasks containing 120 g/l microcrystalline cellulose and 50 g/l CaCO_3_. Selected cultures with highest lactic acid production were used for further evolution in fermenters.

### Analysis of fermentation products

Organic fermentation products lactate, acetate and ethanol were analyzed on a Shimadzu 20A high-performance liquid chromatography (HPLC) system (Shimadzu). Metabolites were separated on a Rezex ROA-Organic Acid H + (8%) 150 × 7.8 mm column (Phenomenex) under isocratic temperature (65 °C) and flow (0.45 ml/min) conditions in 2.5 mM H_2_SO_4_ and then passed through a refractive index (RI) detector (Shimadzu RID-20A). Identification was performed by comparison of retention times with standards.

The yields were calculated assuming maximum formation of 1.67 mol or 2 mol of products (lactate + acetate + ethanol) from 1 mol of C5 or C6 sugar, respectively. The yields were calculated from the ratio between the total molar concentration of products (lactate + acetate + ethanol) formed upon growth on the respective substrates and the expected molar concentration of products at 100% utilization of C5 and C6 sugars in the substrate. Concentrations of glucose, xylose, galactose, arabinose and mannose in all substrates tested were determined according to standard procedure from NREL [41]. Mean values and standard deviations were obtained from three biological replicates.

## Data Availability

The data sets used and/or analyzed during the current study are available from the corresponding author on reasonable request.

## Abbreviations

CBP: Consolidated bioprocessing
MOPS: 3-Morpholinopropanesulfonic acid
NREL: National Renewable Energy Laboratory
HPLC: High-performance liquid chromatography
RI: Refractive index
SCC: Single cell colonies
PLA: Polylactic acid
